# Quantification of nitrate content with FT-NIR technique in lettuce (*Lactuca sativa* L.) variety types: a statistical approach

**DOI:** 10.1007/s13197-020-04442-1

**Published:** 2020-04-23

**Authors:** Ildikó Fruzsina Boros, László Sipos, Noémi Kappel, László Csambalik, Marietta Fodor

**Affiliations:** 1 Department of Vegetable and Mushroom Growing, Faculty of Horticultural Science, Szent István University, 29-43 Villányi út, Budapest, 1118 Hungary; 2 Department of Postharvest Sciences and Sensory Evaluation, Faculty of Food Science, Szent István University, 29-43 Villányi út, Budapest, 1118 Hungary; 3 Department of Ecological and Sustainable Production Systems, Faculty of Horticultural Science, Szent István University, 29-43 Villányi út, Budapest, 1118 Hungary; 4 Department of Applied Chemistry, Faculty of Food Science, Szent István University, 29-43 Villányi út, Budapest, 1118 Hungary

**Keywords:** Lettuce type, Nitrate content, PCA, LDA, NIR, PLS

## Abstract

According to the Commission Regulation (EC) No. 1258/2011, the maximum allowed nitrate content of lettuce is defined within a broad range (2000–5000 mg NO_3_/kg), depending on harvest season and technology. This study focuses on the identification of the differences in nitrate accumulation between lettuce types and varieties, depending on production technology and on the investigation of the application of non-destructive FT-NIR spectroscopy for nitrate quantification, towards widely used UV–Vis spectroscopy.

In the present study, combinations of seasons and technologies (spring × greenhouse, autumn × open field) were employed for the production of types (batavia, butterhead, lollo and oak leaf; both red and green colored); a total of 266 lettuce heads were analyzed. It was found that with standardized technology and conditions, autumn harvested green oak leaf lettuce types accumulated significantly less nitrate, than red oak or lollo leaf types. With spring harvested lettuces, batavia types generally accumulated generally more nitrates than butterhead types. Based on the linear discriminant analysis (LDA) of FT-NIR measurements the four distinct variety types diverge; the lollo type explicitly diverges from batavia and butterhead types. The LDA further revealed, that within lollo and oak leaf variety types, red and green leaved varieties diverge as well. A model was successfully built for the FT-NIR quantification of the nitrate content of lettuce samples (R^2^ = 0.95; RMSEE = 74.4 mg/kg fresh weight; Q^2^ = 0.90; RMSECV = 99.4 mg/kg fresh weight). The developed model is capable of the execution of a fast and non-invasive measurement; the method is suitable for the routine measurement of nitrate content in lettuce.

**Electronic supplementary material:**

The online version of this article (10.1007/s13197-020-04442-1) contains supplementary material, which is available to authorized users.

## Introduction

The statistics of the World Health Organization and the results of international studies show that 73% of human health depends on factors which can be influenced; the most important ones of them are lifestyle, environmental factors and the healthcare system. A balanced diet, as well as nutrient, vitamin and minerals intake have an importance in health management. Vegetable and fruit consumption has a multi-level intermediary role in human health and body weight status, and in the prevention of several diseases (stomach- and intestinal cancer, cardiovascular diseases, certain strokes). In a descending order, the ten most significant risk factors are: smoking, alcoholism, hypertension, obesity, high cholesterol, physical inactivity, high blood glucose concentration, neglecting of fruit and vegetable consumption, occupational diseases, and illegal drug consumption. WHO suggest the daily consumption of 400 g of fresh vegetables and fruits (WHO 2003).

Besides useful nutrients, minerals and vitamins, harmful materials (viruses, bacteria, fungi, heavy metals, pesticides, chemicals, secondary or tertiary metabolites) are taken into the human body as well. Nitrates alone are not particularly dangerous, however, their reaction products require attention. In the presence of oral bacteria nitrates reacts enzymatically with saliva and decompose to nitrites, which further react with secondary and tertiary amino-compounds and can form *N*-nitroso compounds (Lijinski 1999). It is widely accepted, that the amount of nitrates consumed with foodstuffs should be reduced; several sources describe the possible risks of nitrate intake (Santamaria 2006).

Methaemoglobinaemia is a well-known blood-disorder in relation with nitrates. It occurs when the oxygen transporting ability of blood is reduced due to the presence of oxidizing compounds (most commonly nitrates), which react with the Fe(II) ion of blood hemoglobin and form the Fe(III) ion. This conformation is called methemoglobin, which is unable to reversibly bind oxygen. The process can be initiated by oxygen as well, but in a slower way, therefore reductase enzyme is able to revert methemoglobin to hemoglobin (Hall 2015). Adults and children are less exposed to the danger of methaemoglobinaemia than infants. This is because in the blood of the latter the concentration of fetal hemoglobin is higher, which can transform into methemoglobin faster, than non-fetal methemoglobin. At the same time there is less reductase enzyme in the body of infants, which could revert methemoglobin into hemoglobin. In the early development phase of infants, water, and vegetables constitutes a particular risk. Currently there are no medical quick medical methods, which could quantify the levels of nitrates and nitrites directly in the blood. Therefore the amount of methemoglobin is measured from the blood instead (Agency for Toxic Substances and Disease Registry 2011).

Studies have suggested, that 80% of consumed nitrates originate from raw vegetables, 15% comes from drinking water, while 5% is from animal products and cereals (EFSA 2008; Hmelak and Cencic 2013). Nitrates accumulate in the edible parts of vegetables (Liu et al. 2014); leaf and root vegetables contain the highest amount of nitrates (Ahluwalia et al. 2016), out of which spinach and lettuce contains the highest amounts (FSA 2017).

The Joint FAO–WHO Expert Committee of Food Additives (JECFA) and the Scientific Committee on Food of the European Commission defined the acceptable daily intake for nitrates and nitrites: 0–3.7 mg for nitrate ions and 0–0.07 mg nitrite ions per body weight kilograms. The Environmental Protection Agency (EPA) of the United States determined a reference dose of 7.0 mg nitrate ion/body mass kilograms and 0.33 mg nitrite ion/body mass kg (EC 1997; US EPA 2002,2009; ATSDR 2011).

There are other regulations regarding nitrate content, e.g. the Commission Regulation (EC) No. 1258/2011 about setting maximum levels for certain contaminants in foodstuffs. This regulation, besides others, controls the maximum nitrate content for fresh vegetable species [spinach (*Spinacia oleracea*), lettuce (*Lactuca sativa* L.), rucola (*Eruca sativa*, *Diplotaxis* sp., *Brassica tenuifolia*, *Sisymbrium tenuifolium*)], depending on production period, production technology and variety type. Based on this regulation, the maximum limit (mg NO_3_^−^/kg for fresh weight) is 2500–3000 mg/kg in spinach, and 2000–5000 mg/kg in lettuce (EC 2011).

Still, only destructive and time-consuming methods are available in analytical chemistry for the accurate quantification of nitrate content (Itoh et al. 2011). Researchers use several different methodologies for nitrate content measurements: Tamme et al. (2006) use the potentiometric method based on GOST 4228–86 standard, Shokrzadeh et al. (2007) apply the molecular absorption spectrometric method of ISO 6635:1984 standard, Kmecl and Žnidarcic (2015) use a continuous flow analyzer (CFA) according to EN 12014–7 standard, while Campanella et al. (2017) use rapid headspace gas chromatography mass spectrometry (GCeMS) and Ion Chromatography UV–vis (IC-UV).

Having regard to the fact that the amount of nitrates and nitrites are one of the key issues of food safety, and that lettuce stands out in nitrate accumulation among vegetables, it is essential to have analytical methods, which are selective, sensitive, accurate and rapid at the same time. Among simple and cheap methodologies, the application of spectrophotometry (e.g. Griss-method) is still widespread for the quantification of nitrates and nitrites. However, this method is time-consuming and has a low sensitivity. Both the repeatability and stability of chemiluminescence is questionable; electrochemistry has a low sensitivity, therefore is unsuitable for routine application. Chromatography and capillary electrophoresis are very sensitive, but more expensive, than spectroscopy and electrochemical methods. Electro-chemiluminescence could be a suitable measurement, but needs further development. Recently, spectrofluorimetric methods are emphasized due to their simplicity, sensitivity, selectivity, and low costs (Wang et al. 2017).

Little is said about the application of NIR-spectroscopy as a quick analytical quick method. In analytical chemistry, the importance of NIR-spectroscopy has risen in the last two decades. NIR-spectroscopy has in the meanwhile accelerated due to the related theoretical and mechanistic advances (Türker-Kaya and Huck 2017). NIR spectroscopy is a high-performance, low-cost, solvent-free, and non-destructive analytical method (López et al 2017), which is suitable for the analysis of several components of different samples, e.g. for maize variety identification from coated seed (Jia et al. 2015), for peach variety separation (Guo et al. 2016), for the identification of coffee taxons (Mees et al. 2018), for the quality control of meat- (Zamora-Rojas et al. 2011) and of rice (Srivastava et al. 2018), for the characterization of bakery raw materials (particle size, color, protein, dry matter, and moisture content) (Szigedi et al. 2011), for dairy product (fat, protein, lactose content) description (Gonzalez-Martin et al. 2009; Pi et al. 2009), for ingredient analysis of beverages (Newgard 2004), and for monitoring wine fermentation (Di Egidio et al. 2010).

Among quick non-destructive analytical and molecule spectroscopic methods used in food analytics, Fourier Transform Near Infrared spectroscopy (FT-NIR) is the one most widely applied (Pokol 2011). The advantage of FT is the ability to compose accurate and reproducible spectra even from complex samples, thus making identification and quantification becomes possible (McCarthy and Kemeny 2008). When using an FT-NIR spectroscope, the spectrum is not direct, as it is taken by an interferometer; the spectrum is given by the Fourier transformation of the recorded interferogram. Multivariate statistical methods are capable of providing a quantitative estimation as well (PLSR) (Szigedi 2013).

A goal of this study was to identify the differences of nitrate accumulation between lettuce varieties and variety types in different production systems. Another goal was to investigate the application of non-destructive FT-NIR spectroscopy for nitrate quantification towards conventional UV–Vis spectroscopy. Models were built up in order to determine the nitrate content of unknown lettuce samples, using the coherences of UV–Vis and FT-NIR data.

## Materials and methods

### Lettuce samples and sample preparation

Lettuce samples were harvested in 2017 in the phase of heading (d = 25–30 cm); Spring harvested ones were produced in a greenhouse, while autumn harvested ones were collected on an open field. The producer for both harvests was the same, and the samples originated from the Southern region of Great Plains, Hungary. Within a season, all conditions were the same for varieties and variety types. Throughout the investigations, a total of 266 lettuce heads were analyzed. In the spring of 2017, six–six heads per lettuce type (biological parallels) of thirty varieties or variety candidates of butterhead (16) and batavia types (14) were measured. In the autumn five biological parallels per lettuce type of 3 green and 2 red leaved varieties/candidates of butterhead, oak leaf, and lollo types were measured. In each single case, spectra were recorded in five repetitions.

The 2 to 4 lower, injured leaves of lettuce heads, as well as the stalk were removed; heads were cut perpendicularly to the stalk, and halves were further processed. Raw, untreated leaves were used for the measurements. Homogenates were prepared by shredding machines (Hauser 400 W, Russell Hobbs 21,510–56 Aura 350SW, Bosch MMR08A1 400 W) In order to exclude issues arising from sample mixing, devices were rinsed and wiped by paper towels after the homogenization of each samples.

### Determination of nitrate reference data with UV–Vis spectrophotometry

The nitrate contents of samples were determined by the method of Cataldo et al. (1975) following a modified sample preparation (hot extraction, clarification with Carrez solution). Photometric measurements were done on 410 nm wavelength by a Thermo Scientific (Walthman, Massachusetts, USA) recording UV/VIS spectrophotometer. For data evaluation, VISIONpro V2.02 (Thermo Scientific, Walthman, USA) software was used.

### FT-NIR spectroscopic measurement

The homogeneous sample was put into the rotatable quartz sample container (d = 85 mm) in an approximately 2 cm thick layer. The recording was done by a BRUKER MPA™ FT-NIR/NIT (Bruker Optik GmbH Ettlingen, Germany) spectrometer in diffuse reflection mode. The scanning speed of the device is 10 kHz, while its spectral resolution is 8 cm^−1^. The measurement wavelength range was 800–2500 nm (wave number: 12500–4000 cm^−1^). Throughout spectrum recording, 32 sub-spectra are recorded, the average of which is the final spectrum. The optical unit of the device is a Rocksolid interferometer, and the detector is lead-sulfide (PbS). Five spectra were recorded from every sample, while stirring and levelling of the samples happened between the measurements. The water content of lettuce is high (96%), therefore the occurrence of water peaks is expected on the spectrum image. In addition, as the measured component (nitrate) is not infraactive, the estimation graph can be created indirectly, only by statistical tools. For this, a high sample number is desired.

### Statistical analyses

The raw data were evaluated with two different pre-treatments [standard normal variable (SNV), multiplicative scatter correction (MSC)]. For data analysis, multivariate, unsupervised (principal component analysis, PCA) and supervised (linear discriminant analysis, LDA) statistical methods were used. Quantitative forecasting was executed with the PLSR (*partial least squares regression*) statistical method. Statistical analyses were done using Statistica 12.0 (Statsoft Inc., Tulsa, OK, USA), XL-Stat software (Addinsoft, 28 West 27th Street, Suite 503, New York, NY 10001, USA) and OPUS 7.2 (Bruker, Ettlingen, Germany) software packages.

The FT-NIR spectra of 266 lettuce heads were recorded and evaluated by PCA and LDA methods. In order to detect spectral outliers, the dataset was analyzed by PCA.

The LDA was used for the separation of variety types; Statistica 12.0 software was used for the execution of these chemometric methods. Stepwise variable selection was chosen, and validation was executed by random grouping validation.

For the evaluation of nitrate content of lettuce varieties and variety types, the Kruskall–Wallis non-parametric test of XL-Stat software was used on 95% significance level, supplemented with the Dunn pairwise post-hoc test with Bonferroni correction. The setting up of the PLSR based estimation graph capable of quantitative determination happened by OPUS 7.2 software.

## Results

### UV–Vis measurement results of nitrate content.

#### Differences among batavia types

In the case of spring harvested batavia type lettuce varieties, BA_05 and BA_09 diverged significantly from BA_13 and BA_16. No further significant differences were detected among groups regarding nitrate content (Fig. S1, Table S1).

#### Differences among butterhead lettuces

Among spring harvested butterhead lettuces, lowest nitrate contents were given by BU_05 and BU_14, while the highest amount was found in BU_13 variety. BU_01, BU_02, BU_04, BU_05, BU_06, BU_08, BU_09, BU_10, and BU_12 varieties did not differ significantly. The variety BU_13 showed a significantly higher nitrate content, than the varieties BU_03, BU_07 and BU_04, while the variety BU_11 significantly diverged from the varieties BU_03 and BU_14 (Figure S2, Table S2).

#### Differences between batavia and butterhead lettuces

When the nitrate content of butterhead and batavia lettuce varieties was compared, the Kruskal–Wallis test showed significant differences only in a few cases. The nitrate content of BA_16 exceeded significantly that of the varieties BU_03, BA_05, BU_14, BU_07, and BA_09. The varieties BU_03 BA_05 BU_14 resulted in significantly lower values than the variety BA_13. In the other cases, no significant difference could be found (Fig. S3, Table S3).

#### Differences between variety types

**Fig. 1:**
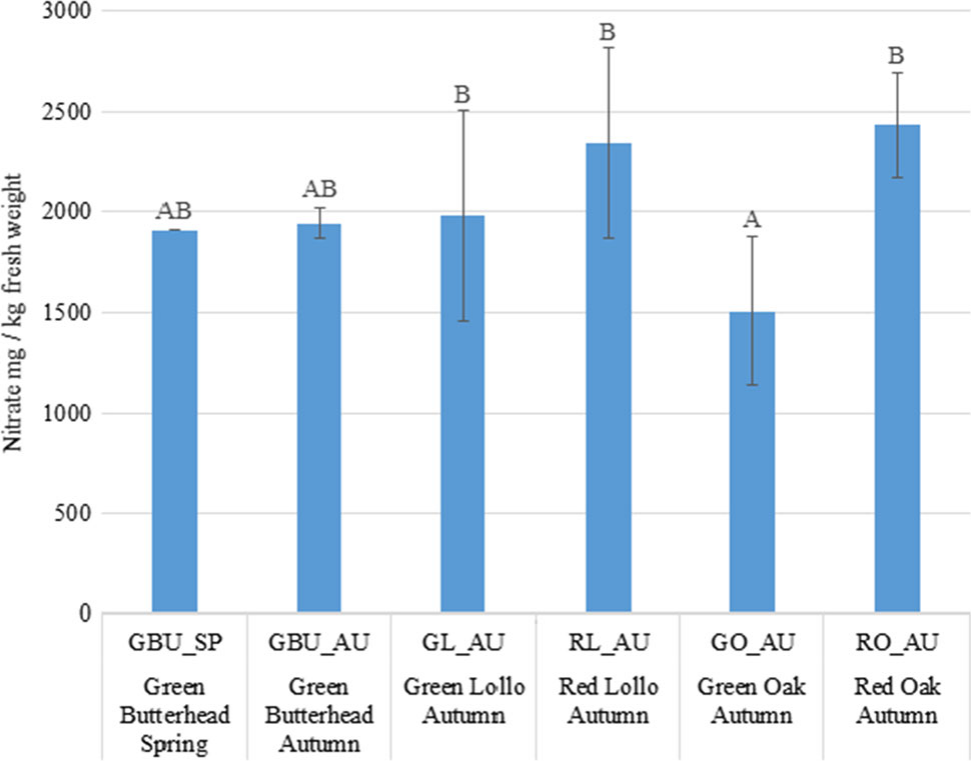
Nitrate content of spring- and autumn harvested lettuce types

The nitrate content of variety type GO_AU was significantly lower than that of variety types RO_AU, GL_AU, and RL_AU. There was no significant difference between the spring and autumn harvested samples of GBU, and these also did not diverge from the other variety types (Fig. 1, Table 1).

**Table 1:** Significant differences between lettuce variety types according to Kruskal–Wallis test statistics (right upper half matrix) and the calculated probability values (italics, left lower half matrix), (Bonferroni-corrected significance level: 0.0033). Bold values indicate significant difference

	Green butterhead (Spring)	Green butterhead (Autumn)	Green lollo (Autumn)	Red lollo (Autumn)	Green Oak leaf (Autumn)	Red Oak leaf (Autumn)
Green butterhead (Spring)	–	1.0667	−3.8667	−22.4000	27.0000	−22.4000
Green butterhead (Autumn)	*0.9110*	–	−4.9333	−23.4667	25.9333	−23.4667
Green lollo (Autumn)	*0.6852*	*0.6050*	–	−18.5333	**30.8667**	−18.5333
Red lollo (Autumn)	*0.0189*	*0.0139*	*0.0520*	–	**49.4000**	−**54.6000**
Green Oak leaf (Autumn)	*0.0046*	*0.0066*	***0.0012***	*** < 0.0001***	–	−**49.4000**
Red Oak leaf (Autumn)	*0.0189*	*0.0139*	*0.0520*	*** < 0.0001***	*** < 0.0001***	–

### Results of FT-NIR spectrum image analysis

**Fig. 2:**
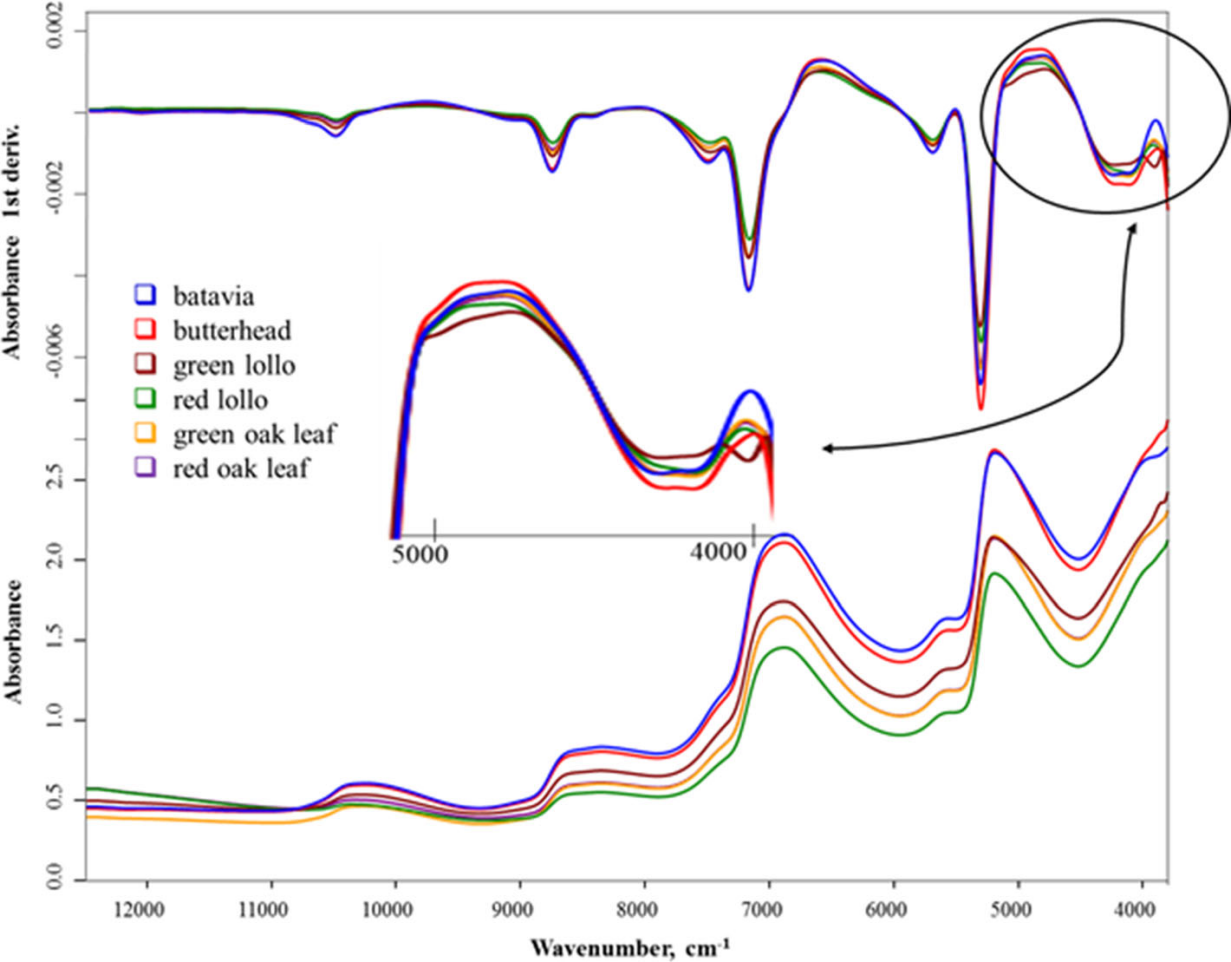
Absorbance spectrum image of each variety types and color variants (average spectrum of all investigated varieties) measured by FT-NIR spectroscope and its relation to each other

Although the homogenization of samples was performed in every case, the first derivative graph transformation was applied in order to reduce spectral differences caused by surface inhomogeneity. After this, spectrum details stand out better, and absorption peaks clearly separate from each other. When investigating the first derivative curve of average spectra (Fig. 2), it is visible that in the wave-number region between 5000 and 3900 cm^−1^ (zoomed area) characteristic differences are shown; here appears the typical absorption of fibers/cellulose, proteins, and carbohydrates, this being the most complex region for NIR analysis.

Peaks visible between 5200–5000 and between 6900–6800 cm^−1^ are the most sensitive combinations and the first overtones of water. Since the water content of the sample is very high, these peaks cover a lot of information. The smaller peak at 10500–10200 cm^−1^ is the overtone of water as well. Differences among variety types are outlined on the spectrum image, however, those of green oak leaf (orange) and red oak leaf (purple) types mostly overlap each other.

### Chemometric analysis of FT-NIR spectrum of lettuce types

The principal component analysis (PCA) without pre-treatment showed that 22 spectra can be considered as outliers; when standard normal variable (SNV), or multiple scattering correction pre-treatments were applied, 16 spectra were outliers, out of which 15 spectra were the same in the case of both pre-treatments (Fig. S4).

**Fig. 3:**
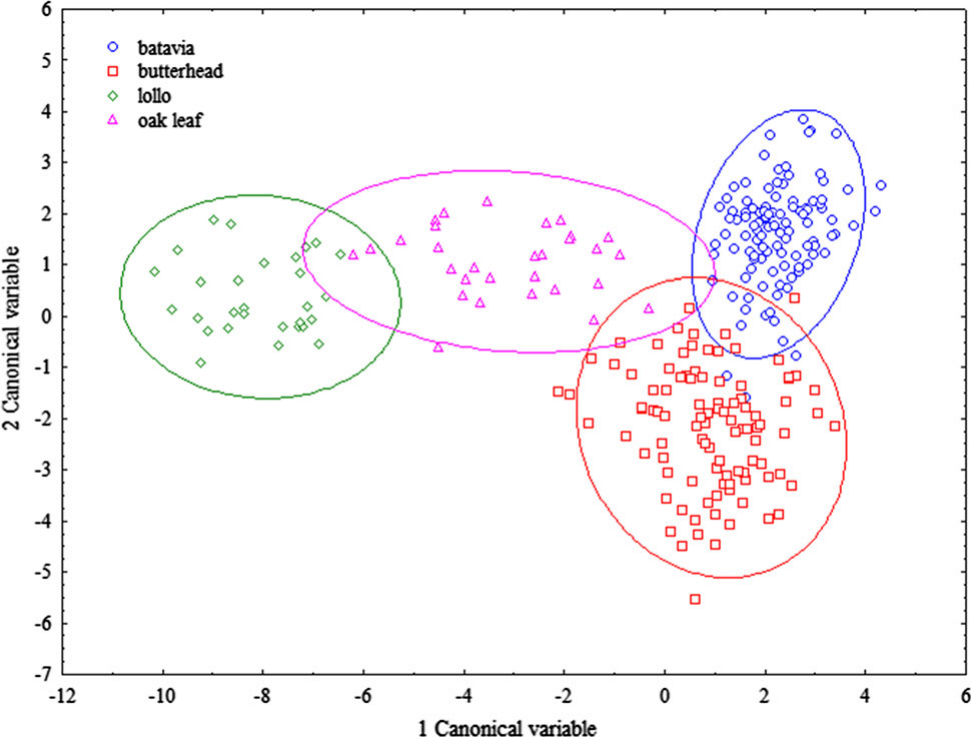
Pattern recognition/classification model of variety types, based on 30 principal component factors, performed by linear discriminant analysis without pre-treatment. Visualization of the second canonical variable was done in the function of the first canonical variable

After the exclusion of spectral outliers, pattern recognition was performed with linear discriminant analysis (LDA); it was found that the studied four variety types diverge from each other, and the lollo type explicitly diverges from batavia and butterhead types (Fig. 3).

The divergence was supported by validation with random grouping, therefore it seems obvious, that groups divided with the LDA method are not created accidentally, but are based on the differences between the measured parameters (Fig. S5).

**Fig. 4:**
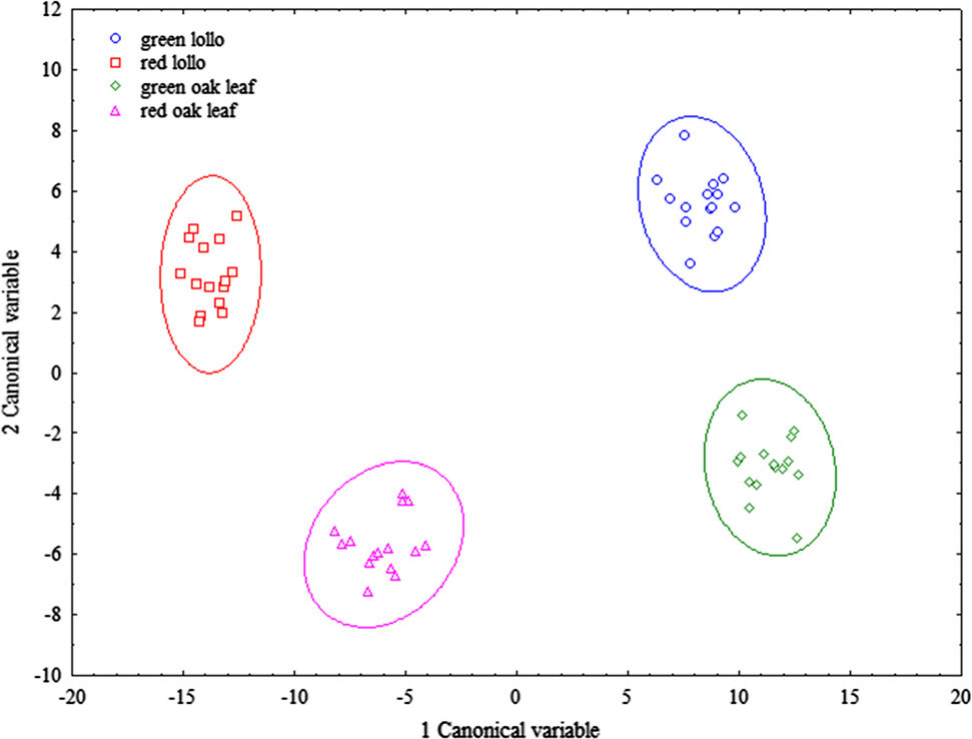
Pattern recognition/classification model of red and green leaved variants of lollo and oak leaf variety types, based on 30 principal component factors, performed by linear discriminant analysis without pre-treatment. Visualization of the second canonical variable was done in the function of the first canonical variable

The LDA further revealed, that within variety types, red and green leaved variants of lollo and oak leaf types definitely diverge from each other as well (Fig. 4), which was also confirmed in this case by the validation with random grouping.

### Characterization of quantitative estimation function

The setup of the PLS estimation function was performed after the exclusion of outliers, with the use of spectral and reference data of 191 samples. For data pre-treatment, MSC was applied. The creation of the model required nine PLS components; the spectral ranges which were taken into consideration for the evaluation were the following: 9558–100 cm^−1^ 7383–5917 cm^−1^ 5199–4467 cm^−1^. The coherence was validated by three-segment cross-validation. The 99.4 mg/kg fresh product average error showed a uniform distribution, and no outstanding sample was found. PLS test validation (1/3 test samples, 2/3 estimation samples) was applied (Q^2^ = 0.90; RMSEP = 114 mg/kg NO_3_^−^). The statistical features of the successful coherence are shown in Table 2.

**Table 2:** Statistical features of the PLS function

Calibration		Validation				RPD	Rank	Range
		Cross validation		Test set validation				
R^2^	RMSEE	Q^2^	RMSECV	Q^2^_T_	RMSEP			
	mg/kg fresh product		mg/kg fresh product		mg/kg fresh product			cm^−1^
0.94	74	0.90	99	0.90	114	3.2	9	9558–8100 7383–5917 5199–4467

*R*^*2*^ Square of determinant coefficient, *RMSEE* Root Mean Square Error of Estimation, *Q*^*2*^ Square of determinant coefficient after cross-validation, *RMSECV* Root-Mean-Square Error of Cross-Validation, *Q*^*2*^_*T*_ Square of determinant coefficient after Test Set validation, *RMSEP* Root-Mean-Square Error of Test Set Validation, *RPD* Residual predictive deviation, *Rank* PLS principal components, *Range* Investigated spectral range

## Discussion

Directive 1258/2011/EC of the European Union defines nitrate content depending on season and production technology. The limit is 5000 mg NO_3_/kg for greenhouse lettuces harvested between 1 October and 31 March, while in the case of iceberg lettuces produced on an open field the maximum allowed amount is 2000 mg NO_3_/kg. The document does not refer to other variety types, nor to color variants. It would be advisable to define this broad range in a variety type-specific manner, in order to reduce food security risks caused by lettuce nitrate intake.

In the present study, a total of 266 lettuce heads were analyzed, in various combinations of seasons and technologies (spring × greenhouse, autumn × open field) and variety types (batavia, butterhead, lollo and oak leaf; both red and green colored).

Based on the UV–Vis measurements run on 410 nm, it can be concluded, that the nitrate content of butterhead lettuce did not show remarkable variation in case of different production technologies and seasons. Regarding autumn harvested lettuces produced in the same environmental conditions, it was found, that green oak leaf types accumulated significantly less nitrates, than red oak or lollo types. In the case of spring harvested samples, batavia types accumulated generally more nitrates, than spring harvested butterhead lettuces.

With the analysis of the image with the spectra, it was proven, that the homogenization of the samples and the first derivation function transformation enhances the inspection of the first derivative graph of spectrum averages, and characteristic differences were found in the region between 5000–3900 cm^−1^ wavenumbers (fibers/cellulose, proteins, carbohydrates). According to this, variety types differ from each other; at the same time, oak leaf types, at the same time, do not diverge from each other. Since the water content of the samples is very high, the peaks at 5200–5000, 6900–6800 cm^−1^ cover a lot of information.

In the pattern recognition/classification model of variety types, based on the linear discriminant analysis (LDA) of FT-NIR measurements, it can be concluded that the four variety types investigated separate from each other, where the lollo type explicitly diverges from batavia and butterhead types. The LDA further revealed, that within variety types, red and green leaved varieties of lollo and oak leaf types diverge as well. The results are also validated by random grouping methodology.

A model was successfully built up for the FT-NIR quantification of nitrate content of lettuce types (R^2^ = 0.95; RMSEE = 74.4 mg/kg fresh product; Q^2^ = 0.90; RMSECV = 99.4 mg/kg fresh product). The developed model is able to execute the measurement in a quick and non-invasive way; the method is suitable for the routine quantification of nitrate content in lettuce samples.

## Electronic supplementary material

Below is the link to the electronic supplementary material.

Supplementary file1 (DOCX 119 kb)

## References

[CR1] Agency for Toxic Substances and Disease Registry (2011) Nitrates and nitrites. Division of Toxicology and Environmental Medicine ToxFAQs. https://www.researchgate.net/profile/Yanick_Simon/post/In_health_risk_assessment_of_nitrates_in_drinking_water_do_nitrates_have_a_threshold_effect_or_not/attachment/59d61da579197b8077978a3c/AS%3A272112726544386%401441888137641/download/ATSDR+Nitrates+and+Nitrites.pdf. Accessed 8 Nov 2018

[CR2] Ahluwalia A, Gladwin M, Coleman GD, Hord N, Howard G, Kim-Shapiro DB, Lajous M, Larsen FJ, Lefer DJ, McClure LA, Nolan BT, Pluta R, Schechter A, Wang CY, Ward MH, Harman JL (2016). Dietary nitrate and the epidemiology of cardiovascular disease: report from a national heart, lung, and blood institute workshop. J Am Heart Assoc.

[CR3] Campanella B, Onor M, Pagliano E (2017). Rapid determination of nitrate in vegetables by gas chromatography mass spectrometry. Anal Chim Acta.

[CR4] Cataldo DA, Maroon M, Schrader LE, Youngs VL (1975). Rapid colorimetric determination of nitrate in plant tissues by nitration of salicylic acid. Commun Soil Sci Plant Anal.

[CR5] Di Egidio V, Sinelli N, Giovanelli G, Moles A, Casiraghi E (2010). NIR and MIR spectroscopy as rapid methods to monitor red wine fermentation. Eur Food Res Technol.

[CR6] European Commision (1997) Opinion on nitrate and nitrite. Reports of the Scientific Committee for Food (SCF) No 197 38th Series:1–33. https://ec.europa.eu/food/fs/sc/scf/reports/scf_reports_38.pdf. Accessed 8 Nov 2018

[CR7] Commission European (2011). COMMISSION REGULATION (EU) No 1258/2011 of 2 December 2011 amending Regulation (EC) No 1881/2006 as regards maximum levels for nitrates in foodstuffs. Official J Eur Union.

[CR8] European Food Safety Authority (2008). Nitrate in vegetables. Scientific opinion of the panel on contaminants in the food chain. EFSA J.

[CR10] Food Standards Agency (2017) Nitrate monitoring in spinach and lettuce—surveillance programme. https://www.food.gov.uk/research/research-projects/nitrate-monitoring-in-spinach-and-lettuce-surveillance-programme. Accessed 8 Nov 2018

[CR11] Gonzalez-Martin I, Hernandez-Hierro JM, Vivar-Quintana A, Revilla I, Gonzalez-Perez C (2009). The application of near infrared spectroscopy technology and a remote reflectance fibre-optic probe for the determination of peptides in cheeses (cow's, ewe's and goat's) with different ripening times. Food Chem.

[CR12] Guo W, Gu J, Liu D, Shang L (2016). Peach variety identification using near-infrared diffuse reflectance spectroscopy. Comput Electron Agric.

[CR9] Hall JE (2015). Guyton and hall textbook of medical physiology.

[CR13] Hmelak Gorenjak A, Cencic A (2013). Nitrate in vegetables and their impact on human health. A review Acta Aliment.

[CR14] Itoh H, Tomita H, Uno Y, Shiraishi N (2011) Development of Method for Non-destructive Measurement of Nitrate Concentration in Vegetable Leaves by Near-infrared Spectroscopy. 18th IFAC World Congress. Milano (Italy), August 28 - September 2. doi: 10.3182/20110828-6-IT-1002.00738

[CR15] Jia S, An D, Liu Z, Gu J, Li S, Zhang X, Zhu D, Guo T, Yan Y (2015). Variety identification method of coated maize seeds based on near-infrared spectroscopy and chemometrics. J Cereal Sci.

[CR16] Kmecl V, Žnidarcic D (2015). Accreditation of the analytical method used for nitrate determination in vegetables. Arch Biol Sci.

[CR17] Lijinski W (1999). N-Nitroso compounds in the diet. Mutat Res/Gen Toxicol Environ Mutagen.

[CR18] Liu CW, Sung Y, Chen BC, Lai HY (2014). Effects of nitrogen fertilizers on the growth and nitrate content of lettuce (Lactuca sativa L.). Int J Environ Res Public Health.

[CR19] López MG, Garcia-González AS, Franco-Robles E (2017) Carbohydrate Analysis by NIRS-Chemometrics. Chapter 4. In: Kyprianidis K and Skvaril J (eds.) (2017) Developments in Near-Infrared Spectroscopy, IntechOpen, London, UK 10.5772/62932

[CR20] McCarthy WJ, Kemeny GJ (2008) Fourier Transform Specthrophotometers in the Near-Infrared (chapter 5) In: Burns DA, Ciurczak EW (eds.) Handbook of Near-Infrared Analysis, 3rd Edition. Practical Spectroscopy vol. 35 CRC Press, USA, Boca Raton

[CR21] Mees C, Souard F, Delporte C, Deconinck E, Stoffelen P, Stévigny C, Kaufmann JM, De Braekeleer K (2018). Identification of coffee leaves using FT-NIR spectroscopy and SIMCA. Talanta.

[CR22] Newgard EC (2004) Near-infrared spectroscopy for analysis of agricultural material. https://www.researchgate.net/publication/253209021_Near-Infrared_Spectroscopy_for_Analysis_of_Agricultural_Material. Accessed 6 Nov 2018

[CR23] Pi F, Shinzawa H, Ozaki Y, Han D (2009). Non-destructive determination of components in processed cheese slice wrapped with a polyethylene film using near-infrared spectroscopy and chemometrics. Int Dairy J.

[CR24] Pokol Gy (2011). Analitikai Kémia.

[CR25] Shokrzadeh M, Shokravie M, Saeedi Saravi SS (2007). The measurement of nitrate and nitrite content in leek and spinach sampled from central cities of Mazandaran State of Iran. World Appl Sci J.

[CR26] Srivastava S, Mishra G, Mishra NM (2018). FTNIR-A Robust diagnostic tool for the rapid detection of *Rhyzopertha dominica* and *Sitophilus oryzae* infestation and quality changes in stored rice grains. Food Bioproc Tech.

[CR27] Szigedi T (2013) Módszerfejlesztés Fourier-transzformációs közeli infravörös technika (FT-NIR) alkalmazási körének kibővítésére élelmiszeripari mintákon. Doctoral thesis. 10.14267/phd.2014009

[CR28] Szigedi T, Dernovics M, Fodor M (2011). Determination of protein, lipid and sugar contents of bakery products by using fourier-transform near infrared spectroscopy. Acta Aliment.

[CR29] Tamme T, Reinik M, Roasto M, Juhkam K, Tenno T, Kiis A (2006). Nitrates and nitrites in vegetables and vegetable-based products and their intakes by the Estonian population. Food Addit Contam.

[CR30] Türker-Kaya S, Huck CW (2017). A review of mid-infrared and near-infrared imaging: principles, concepts and applications in plant tissue analysis. Molecules.

[CR31] US Environmental Protection Agency (2002) Integrated Risk Information System (IRIS) database. Nitrate (CASRN 14797-55-8). Washington DC. https://cfpub.epa.gov/ncea/iris/iris_documents/documents/subst/0076_summary.pdf. Accessed 8 Nov 2018

[CR32] US Environmental Protection Agency (2009) National Primary Drinking Water Regulations (EPA 816-F-09-004) https://www.epa.gov/sites/production/files/2016-06/documents/npwdr_complete_table.pdf. Accessed 8 Nov 2018

[CR33] Wang QH, Yu LJ, Liu Y, Lin L, Lu RG, Zhu JP, He L, Lu ZL (2017). Methods for the detection and determination of nitrite and nitrate: a review. Talanta.

[CR34] WHO (2003) Diet, nutrition and the prevention of chronic diseases. Report of a Joint FAO/WHO Expert Consultation. Geneva, 28 January–1 February 2002. (WHO technical report series, No.: 916). 12768890

[CR35] Zamora-Rojas E, Pérez-Marín D, De Pedro-Sanz E, Guerrero-Ginel JE, Garrido-Varo A (2011). In-situ Iberian pig carcass classification using a micro-electro-mechanical system (MEMS)-based near infrared (NIR) spectrometer. Meat Sci.

[CR36] Santamaria P (2006). Nitrate in vegetables: toxicity, content, intake and EC regulation. J Sci Food Agric.

