# Supplementary material for: Quantification of nitrate content with FT-NIR technique in lettuce (Lactuca sativa L.) variety types: a statistical approach
Source: J Food Sci Technol. 2020 Apr 23;57(11):4084–91. doi: 10.1007/s13197-020-04442-1 (PMC7520474; doi:10.1007/s13197-020-04442-1)
Supplement: Supplementary file 1 — Supplementary file1 (DOCX 119 kb) [file 13197_2020_4442_MOESM1_ESM.docx]

**Supplementary material**

**Figures**

**List of figures**

**Fig. S1** Nitrate content of batavia (BA) types

**Fig. S2** Nitrate content of butterhead (BU) lettuces

**Fig. S3** The nitrate content of the investigated (batavia (BA) and butterhead (BU)) lettuce varieties

**Fig. S4** Searching spectral outlying samples (extreme values) with principal component analysis without pre-treatment (PC1=79.5%, PC2=19.5%). Blue line indicates the 95% confidence interval. Outlying samples are marked with red circles.

**Fig. S5** Validation of pattern recognition/classification model of variety types with random grouping. Visualization of the second canonical variable was done in the function of the first canonical variable

Fig. S1


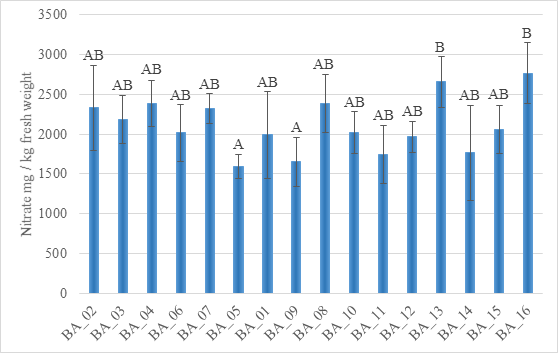


Fig. S2


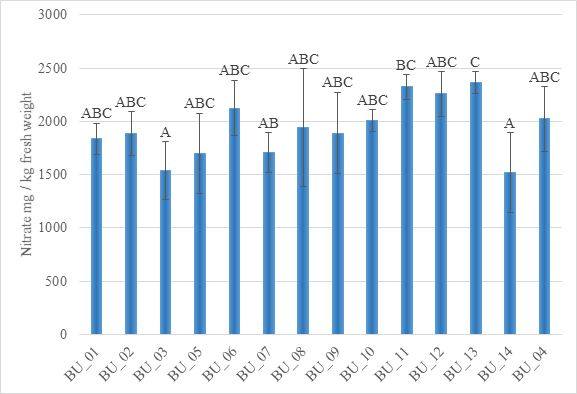


Fig. S3
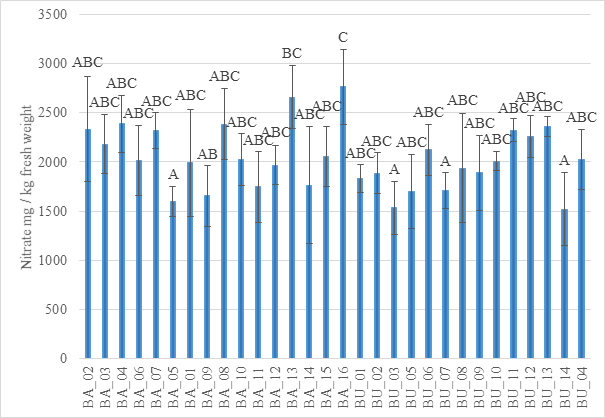


Fig. S4
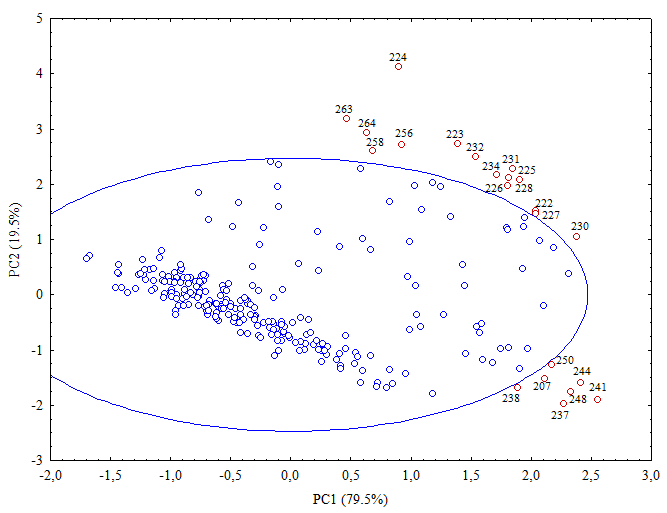


Fig. S5
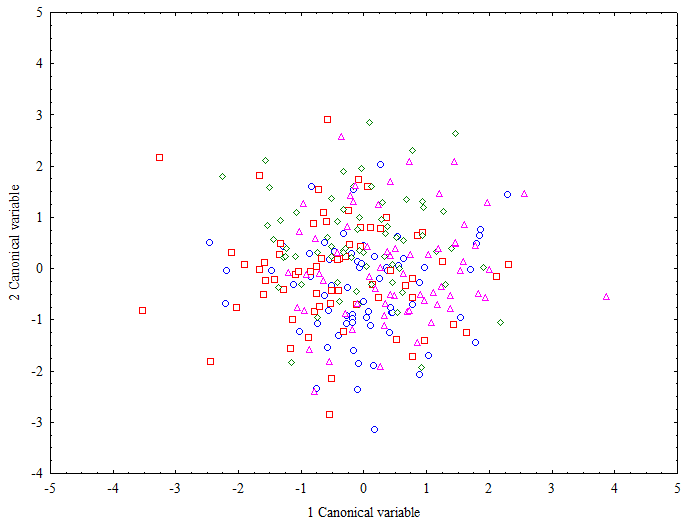


**Tables**

**Table S1** Significant differences between batavia (BA) lettuce varieties according to Kruskal-Wallis test statistics (right upper half matrix) and the calculated probability values (*italics*, left lower half matrix), (Bonferroni-corrected significance level: 0.0004). **Bold** values indicate significant difference.

|  | BA_02 | BA_03 | BA_04 | BA_06 | BA_07 | BA_05 | BA_01 | BA_09 | BA_08 | BA_10 | BA_11 | BA_12 | BA_13 | BA_14 | BA_15 | BA_16 |
| --- | --- | --- | --- | --- | --- | --- | --- | --- | --- | --- | --- | --- | --- | --- | --- | --- |
| BA_02 | – | 6.6667 | -7.7500 | 16.5000 | -3.6667 | 44.8333 | 19.3333 | 38.0833 | -7.1667 | 15.6667 | 32.3333 | 19.5833 | -20.0833 | 27.1667 | 13.1667 | -24.0000 |
| BA_03 | *0.6785* | – | -14.4167 | 9.8333 | -10.3333 | 38.1667 | 12.6667 | 31.4167 | -13.8333 | 9.0000 | 25.6667 | 12.9167 | -26.7500 | 20.5000 | 6.5000 | -30.6667 |
| BA_04 | *0.6299* | *0.3700* | – | 24.2500 | 4.0833 | 52.5833 | 27.0833 | 45.8333 | 0.5833 | 23.4167 | 40.0833 | 27.3333 | -12.3333 | 34.9167 | 20.9167 | -16.2500 |
| BA_06 | *0.3049* | *0.5409* | *0.1316* | – | -20.1667 | 28.3333 | 2.8333 | 21.5833 | -23.6667 | -0.8333 | 15.8333 | 3.0833 | -36.5833 | 10.6667 | -3.3333 | -40.5000 |
| BA_07 | *0.8197* | *0.5205* | *0.7996* | *0.2099* | – | 48.5000 | 23.0000 | 41.7500 | -3.5000 | 19.3333 | 36.0000 | 23.2500 | -16.4167 | 30.8333 | 16.8333 | -20.3333 |
| BA_05 | *0.0053* | *0.0176* | *0.0011* | *0.0781* | *0.0026* | – | -25.5000 | -6.7500 | -52.0000 | -29.1667 | -12.5000 | -25.2500 | -64.9167 | -17.6667 | -31.6667 | -68.8333 |
| BA_01 | *0.2293* | *0.4309* | *0.0922* | *0.8602* | *0.1527* | *0.1128* | – | 18.7500 | -26.5000 | -3.6667 | 13.0000 | 0.2500 | -39.4167 | 7.8333 | -6.1667 | -43.3333 |
| BA_09 | *0.0179* | *0.0508* | *0.0044* | *0.1796* | *0.0094* | *0.6747* | *0.2437* | – | -45.2500 | -22.4167 | -5.7500 | -18.5000 | -58.1667 | -10.9167 | -24.9167 | -62.0833 |
| BA_08 | *0.6559* | *0.3897* | *0.9711* | *0.1411* | *0.8277* | *0.0012* | *0.0994* | *0.0049* | – | 22.8333 | 39.5000 | 26.7500 | -12.9167 | 34.3333 | 20.3333 | -16.8333 |
| BA_10 | *0.3300* | *0.5758* | *0.1454* | *0.9587* | *0.2293* | *0.0698* | *0.8197* | *0.1634* | *0.1557* | – | 16.6667 | 3.9167 | -35.7500 | 11.5000 | -2.5000 | -39.6667 |
| BA_11 | *0.0444* | *0.1105* | *0.0127* | *0.3249* | *0.0252* | *0.4370* | *0.4189* | *0.7207* | *0.0140* | *0.3001* | – | -12.7500 | -52.4167 | -5.1667 | -19.1667 | -56.3333 |
| BA_12 | *0.2234* | *0.4219* | *0.0892* | *0.8480* | *0.1483* | *0.1164* | *0.9876* | *0.2500* | *0.0963* | *0.8076* | *0.4279* | – | -39.6667 | 7.5833 | -6.4167 | -43.5833 |
| BA_13 | *0.2118* | *0.0963* | *0.4432* | *0.0229* | *0.3074* | ***< 0.0001*** | *0.0143* | ***0.0003*** | *0.4219* | *0.0262* | *0.0011* | *0.0136* | – | 47.2500 | 33.2500 | -3.9167 |
| BA_14 | *0.0912* | *0.2024* | *0.0299* | *0.5072* | *0.0552* | *0.2720* | *0.6262* | *0.4973* | *0.0328* | *0.4746* | *0.7480* | *0.6373* | *0.0033* | – | -14.0000 | -51.1667 |
| BA_15 | *0.4130* | *0.6861* | *0.1934* | *0.8358* | *0.2953* | *0.0490* | *0.7014* | *0.1213* | *0.2061* | *0.8765* | *0.2334* | *0.6899* | *0.0387* | *0.3840* | – | -37.1667 |
| BA_16 | *0.1356* | *0.0565* | *0.3123* | *0.0118* | *0.2061* | ***< 0.0001*** | *0.0071* | ***0.0001*** | *0.2953* | *0.0136* | *0.0005* | *0.0067* | *0.8076* | *0.0015* | *0.0208* | – |

**Table S2** Significant differences between butterhead (BU) lettuce varieties according to Kruskal-Wallis test statistics (right upper half matrix) and the calculated probability values (italics*,* left lower half matrix), (Bonferroni-corrected significance level: 0.0005)*.* **Bold** values indicate significant difference.

|  | BU_01 | BU_02 | BU_03 | BU_05 | BU_06 | BU_07 | BU_08 | BU_09 | BU_10 | BU_11 | BU_12 | BU_13 | BU_14 | BU_04 |
| --- | --- | --- | --- | --- | --- | --- | --- | --- | --- | --- | --- | --- | --- | --- |
| BU_01 | – | -5.3333 | 13.6667 | 1.5000 | -23.6667 | 8.0000 | -15.6667 | -10.5000 | -16.6667 | -40.0000 | -34.5000 | -42.6667 | 12.3333 | -16.8333 |
| BU_02 | *0.7049* | – | 19.0000 | 6.8333 | -18.3333 | 13.3333 | -10.3333 | -5.1667 | -11.3333 | -34.6667 | -29.1667 | -37.3333 | 17.6667 | -11.5000 |
| BU_03 | *0.3318* | *0.1773* | – | -12.1667 | -37.3333 | -5.6667 | -29.3333 | -24.1667 | -30.3333 | -53.6667 | -48.1667 | -56.3333 | -1.3333 | -30.5000 |
| BU_05 | *0.9152* | *0.6275* | *0.3876* | – | -25.1667 | 6.5000 | -17.1667 | -12.0000 | -18.1667 | -41.5000 | -36.0000 | -44.1667 | 10.8333 | -18.3333 |
| BU_06 | *0.0929* | *0.1930* | *0.0080* | *0.0739* | – | 31.6667 | 8.0000 | 13.1667 | 7.0000 | -16.3333 | -10.8333 | -19.0000 | 36.0000 | 6.8333 |
| BU_07 | *0.5700* | *0.3437* | *0.6874* | *0.6444* | *0.0245* | – | -23.6667 | -18.5000 | -24.6667 | -48.0000 | -42.5000 | -50.6667 | 4.3333 | -24.8333 |
| BU_08 | *0.2659* | *0.4631* | *0.0373* | *0.2228* | *0.5700* | *0.0929* | – | 5.1667 | -1.0000 | -24.3333 | -18.8333 | -27.0000 | 28.0000 | -1.1667 |
| BU_09 | *0.4559* | *0.7137* | *0.0862* | *0.3942* | *0.3498* | *0.1890* | *0.7137* | – | -6.1667 | -29.5000 | -24.0000 | -32.1667 | 22.8333 | -6.3333 |
| BU_10 | *0.2366* | *0.4210* | *0.0312* | *0.1971* | *0.6191* | *0.0799* | *0.9434* | *0.6615* | – | -23.3333 | -17.8333 | -26.0000 | 29.0000 | -0.1667 |
| BU_11 | *0.0045* | *0.0138* | ***0.0001*** | *0.0032* | *0.2461* | *0.0007* | *0.0840* | *0.0362* | *0.0975* | – | 5.5000 | -2.6667 | 52.3333 | 23.1667 |
| BU_12 | *0.0143* | *0.0383* | *0.0006* | *0.0106* | *0.4417* | *0.0025* | *0.1811* | *0.0883* | *0.2054* | *0.6961* | – | -8.1667 | 46.8333 | 17.6667 |
| BU_13 | *0.0024* | *0.0080* | ***< 0.0001*** | *0.0017* | *0.1773* | ***0.0003*** | *0.0552* | *0.0224* | *0.0649* | *0.8498* | *0.5620* | – | 55.0000 | 25.8333 |
| BU_14 | *0.3812* | *0.2097* | *0.9246* | *0.4417* | *0.0106* | *0.7583* | *0.0468* | *0.1049* | *0.0395* | ***0.0002*** | *0.0009* | ***< 0.0001*** | – | -29.1667 |
| BU_04 | *0.2320* | *0.4142* | *0.0303* | *0.1930* | *0.6275* | *0.0778* | *0.9340* | *0.6529* | *0.9906* | *0.1000* | *0.2097* | *0.0666* | *0.0383* | – |

**Table S3** Significant differences between batavia (BA) and butterhead (BU) lettuce varieties according to Kruskal-Wallis test statistics (right upper half matrix) and the calculated probability values (*italics*, left lower half matrix), (Bonferroni-corrected significance level: 0.0001). **Bold** values indicate significant difference.

|  | BA_02 | BA_03 | BA_04 | BA_06 | BA_07 | BA_05 | BA_01 | BA_09 | BA_08 | BA_10 | BA_11 | BA_12 | BA_13 | BA_14 | BA_15 |
| --- | --- | --- | --- | --- | --- | --- | --- | --- | --- | --- | --- | --- | --- | --- | --- |
| BA_02 | – | 7.0000 | -19.0000 | 27.6667 | -14.7500 | 87.0000 | 37.1667 | 72.9167 | -17.6667 | 28.6667 | 60.9167 | 36.9167 | -40.2500 | 51.0000 | 26.7500 |
| BA_03 | *0.8160* | – | -26.0000 | 20.6667 | -21.7500 | 80.0000 | 30.1667 | 65.9167 | -24.6667 | 21.6667 | 53.9167 | 29.9167 | -47.2500 | 44.0000 | 19.7500 |
| BA_04 | *0.5277* | *0.3874* | – | 46.6667 | 4.2500 | 106.0000 | 56.1667 | 91.9167 | 1.3333 | 47.6667 | 79.9167 | 55.9167 | -21.2500 | 70.0000 | 45.7500 |
| BA_06 | *0.3577* | *0.4921* | *0.1208* | – | -42.4167 | 59.3333 | 9.5000 | 45.2500 | -45.3333 | 1.0000 | 33.2500 | 9.2500 | -67.9167 | 23.3333 | -0.9167 |
| BA_07 | *0.6239* | *0.4697* | *0.8877* | *0.1585* | – | 101.7500 | 51.9167 | 87.6667 | -2.9167 | 43.4167 | 75.6667 | 51.6667 | -25.5000 | 65.7500 | 41.5000 |
| BA_05 | *0.0038* | *0.0078* | *0.0004* | *0.0486* | *0.0007* | – | -49.8333 | -14.0833 | -104.6667 | -58.3333 | -26.0833 | -50.0833 | -127.2500 | -36.0000 | -60.2500 |
| BA_01 | *0.2167* | *0.3160* | *0.0619* | *0.7522* | *0.0844* | *0.0976* | – | 35.7500 | -54.8333 | -8.5000 | 23.7500 | -0.2500 | -77.4167 | 13.8333 | -10.4167 |
| BA_09 | *0.0154* | *0.0284* | *0.0022* | *0.1325* | *0.0036* | *0.6397* | *0.2347* | – | -90.5833 | -44.2500 | -12.0000 | -36.0000 | -113.1667 | -21.9167 | -46.1667 |
| BA_08 | *0.5570* | *0.4122* | *0.9646* | *0.1318* | *0.9228* | *0.0005* | *0.0683* | *0.0026* | – | 46.3333 | 78.5833 | 54.5833 | -22.5833 | 68.6667 | 44.4167 |
| BA_10 | *0.3406* | *0.4714* | *0.1131* | *0.9735* | *0.1490* | *0.0525* | *0.7775* | *0.1413* | *0.1235* | – | 32.2500 | 8.2500 | -68.9167 | 22.3333 | -1.9167 |
| BA_11 | *0.0429* | *0.0731* | *0.0079* | *0.2690* | *0.0119* | *0.3859* | *0.4298* | *0.6900* | *0.0090* | *0.2837* | – | -24.0000 | -101.1667 | -9.9167 | -34.1667 |
| BA_12 | *0.2198* | *0.3200* | *0.0631* | *0.7585* | *0.0859* | *0.0959* | *0.9934* | *0.2314* | *0.0696* | *0.7839* | *0.4250* | – | -77.1667 | 14.0833 | -10.1667 |
| BA_13 | *0.1809* | *0.1163* | *0.4800* | *0.0240* | *0.3966* | *< 0.0001* | *0.0101* | *0.0002* | *0.4528* | *0.0220* | *0.0008* | *0.0103* | – | 91.2500 | 67.0000 |
| BA_14 | *0.0900* | *0.1436* | *0.0200* | *0.4380* | *0.0288* | *0.2314* | *0.6456* | *0.4663* | *0.0225* | *0.4579* | *0.7417* | *0.6397* | *0.0024* | – | -24.2500 |
| BA_15 | *0.3739* | *0.5115* | *0.1283* | *0.9757* | *0.1677* | *0.0452* | *0.7291* | *0.1249* | *0.1398* | *0.9492* | *0.2561* | *0.7354* | *0.0259* | *0.4202* | – |
| BA_16 | *0.1391* | *0.0869* | *0.3966* | *0.0164* | *0.3227* | *< 0.0001* | *0.0066* | *< 0.0001* | *0.3724* | *0.0150* | *0.0005* | *0.0068* | *0.8877* | *0.0015* | *0.0179* |
| BU_01 | *0.0589* | *0.0976* | *0.0117* | *0.3323* | *0.0173* | *0.3160* | *0.5133* | *0.5929* | *0.0133* | *0.3491* | *0.8920* | *0.5079* | *0.0013* | *0.8462* | *0.3173* |
| BU_02 | *0.1004* | *0.1585* | *0.0230* | *0.4697* | *0.0329* | *0.2115* | *0.6839* | *0.4347* | *0.0258* | *0.4903* | *0.7023* | *0.6778* | *0.0029* | *0.9580* | *0.4512* |
| BU_03 | *0.0036* | *0.0073* | *0.0004* | *0.0461* | *0.0007* | *0.9823* | *0.0932* | *0.6239* | *0.0005* | *0.0499* | *0.3739* | *0.0916* | *< 0.0001* | *0.2229* | *0.0429* |
| BU_05 | *0.0318* | *0.0556* | *0.0055* | *0.2198* | *0.0084* | *0.4562* | *0.3621* | *0.7818* | *0.0063* | *0.2325* | *0.9030* | *0.3577* | *0.0005* | *0.6516* | *0.2085* |
| BU_06 | *0.6377* | *0.8117* | *0.2702* | *0.6536* | *0.3364* | *0.0155* | *0.4445* | *0.0508* | *0.2900* | *0.6298* | *0.1202* | *0.4495* | *0.0705* | *0.2208* | *0.6757* |
| BU_07 | *0.0141* | *0.0263* | *0.0020* | *0.1249* | *0.0032* | *0.6616* | *0.2229* | *0.9757* | *0.0024* | *0.1333* | *0.6677* | *0.2198* | *0.0001* | *0.4478* | *0.1176* |
| BU_08 | *0.3213* | *0.4478* | *0.1045* | *0.9426* | *0.1383* | *0.0574* | *0.8074* | *0.1521* | *0.1143* | *0.9691* | *0.3015* | *0.8139* | *0.0198* | *0.4817* | *0.9184* |
| BU_09 | *0.1836* | *0.2727* | *0.0499* | *0.6818* | *0.0688* | *0.1182* | *0.9250* | *0.2739* | *0.0552* | *0.7064* | *0.4869* | *0.9184* | *0.0076* | *0.7146* | *0.6596* |
| BU_10 | *0.3491* | *0.4817* | *0.1169* | *0.9867* | *0.1537* | *0.0505* | *0.7648* | *0.1369* | *0.1276* | *0.9867* | *0.2763* | *0.7712* | *0.0230* | *0.4478* | *0.9624* |
| BU_11 | *0.6141* | *0.4612* | *0.8986* | *0.1545* | *0.9889* | *0.0007* | *0.0819* | *0.0034* | *0.9338* | *0.1451* | *0.0114* | *0.0834* | *0.4044* | *0.0278* | *0.1635* |
| BU_12 | *0.8636* | *0.6859* | *0.6456* | *0.2751* | *0.7501* | *0.0022* | *0.1594* | *0.0094* | *0.6778* | *0.2607* | *0.0280* | *0.1618* | *0.2435* | *0.0619* | *0.2887* |
| BU_13 | *0.4903* | *0.3563* | *0.9536* | *0.1075* | *0.8419* | *0.0003* | *0.0542* | *0.0018* | *0.9184* | *0.1004* | *0.0066* | *0.0552* | *0.5169* | *0.0171* | *0.1143* |
| BU_14 | *0.0052* | *0.0105* | *0.0006* | *0.0611* | *0.0010* | *0.9206* | *0.1195* | *0.7126* | *0.0007* | *0.0659* | *0.4429* | *0.1176* | *< 0.0001* | *0.2727* | *0.0570* |
| BU_04 | *0.3563* | *0.4903* | *0.1202* | *0.9978* | *0.1577* | *0.0489* | *0.7543* | *0.1333* | *0.1311* | *0.9757* | *0.2702* | *0.7606* | *0.0238* | *0.4396* | *0.9735* |

**Table S3** (continued): Significant differences between batavia (BA) and butterhead (BU) lettuce varieties according to Kruskal-Wallis test statistics (right upper half matrix) and the calculated probability values (*italics*, left lower half matrix), (Bonferroni-corrected significance level: 0.0001). **Bold** values indicate significant difference.

| BA_16 | BU_01 | BU_02 | BU_03 | BU_05 | BU_06 | BU_07 | BU_08 | BU_09 | BU_10 | BU_11 | BU_12 | BU_13 | BU_14 | BU_04 |
| --- | --- | --- | --- | --- | --- | --- | --- | --- | --- | --- | --- | --- | --- | --- |
| -44.5000 | 56.8333 | 49.4167 | 87.6667 | 64.5833 | 14.1667 | 73.8333 | 29.8333 | 40.0000 | 28.1667 | -15.1667 | -5.1667 | -20.7500 | 84.0000 | 27.7500 |
| -51.5000 | 49.8333 | 42.4167 | 80.6667 | 57.5833 | 7.1667 | 66.8333 | 22.8333 | 33.0000 | 21.1667 | -22.1667 | -12.1667 | -27.7500 | 77.0000 | 20.7500 |
| -25.5000 | 75.8333 | 68.4167 | 106.6667 | 83.5833 | 33.1667 | 92.8333 | 48.8333 | 59.0000 | 47.1667 | 3.8333 | 13.8333 | -1.7500 | 103.0000 | 46.7500 |
| -72.1667 | 29.1667 | 21.7500 | 60.0000 | 36.9167 | -13.5000 | 46.1667 | 2.1667 | 12.3333 | 0.5000 | -42.8333 | -32.8333 | -48.4167 | 56.3333 | 0.0833 |
| -29.7500 | 71.5833 | 64.1667 | 102.4167 | 79.3333 | 28.9167 | 88.5833 | 44.5833 | 54.7500 | 42.9167 | -0.4167 | 9.5833 | -6.0000 | 98.7500 | 42.5000 |
| -131.5000 | -30.1667 | -37.5833 | 0.6667 | -22.4167 | -72.8333 | -13.1667 | -57.1667 | -47.0000 | -58.8333 | -102.1667 | -92.1667 | -107.7500 | -3.0000 | -59.2500 |
| -81.6667 | 19.6667 | 12.2500 | 50.5000 | 27.4167 | -23.0000 | 36.6667 | -7.3333 | 2.8333 | -9.0000 | -52.3333 | -42.3333 | -57.9167 | 46.8333 | -9.4167 |
| -117.4167 | -16.0833 | -23.5000 | 14.7500 | -8.3333 | -58.7500 | 0.9167 | -43.0833 | -32.9167 | -44.7500 | -88.0833 | -78.0833 | -93.6667 | 11.0833 | -45.1667 |
| -26.8333 | 74.5000 | 67.0833 | 105.3333 | 82.2500 | 31.8333 | 91.5000 | 47.5000 | 57.6667 | 45.8333 | 2.5000 | 12.5000 | -3.0833 | 101.6667 | 45.4167 |
| -73.1667 | 28.1667 | 20.7500 | 59.0000 | 35.9167 | -14.5000 | 45.1667 | 1.1667 | 11.3333 | -0.5000 | -43.8333 | -33.8333 | -49.4167 | 55.3333 | -0.9167 |
| -105.4167 | -4.0833 | -11.5000 | 26.7500 | 3.6667 | -46.7500 | 12.9167 | -31.0833 | -20.9167 | -32.7500 | -76.0833 | -66.0833 | -81.6667 | 23.0833 | -33.1667 |
| -81.4167 | 19.9167 | 12.5000 | 50.7500 | 27.6667 | -22.7500 | 36.9167 | -7.0833 | 3.0833 | -8.7500 | -52.0833 | -42.0833 | -57.6667 | 47.0833 | -9.1667 |
| -4.2500 | 97.0833 | 89.6667 | 127.9167 | 104.8333 | 54.4167 | 114.0833 | 70.0833 | 80.2500 | 68.4167 | 25.0833 | 35.0833 | 19.5000 | 124.2500 | 68.0000 |
| -95.5000 | 5.8333 | -1.5833 | 36.6667 | 13.5833 | -36.8333 | 22.8333 | -21.1667 | -11.0000 | -22.8333 | -66.1667 | -56.1667 | -71.7500 | 33.0000 | -23.2500 |
| -71.2500 | 30.0833 | 22.6667 | 60.9167 | 37.8333 | -12.5833 | 47.0833 | 3.0833 | 13.2500 | 1.4167 | -41.9167 | -31.9167 | -47.5000 | 57.2500 | 1.0000 |
| – | 101.3333 | 93.9167 | 132.1667 | 109.0833 | 58.6667 | 118.3333 | 74.3333 | 84.5000 | 72.6667 | 29.3333 | 39.3333 | 23.7500 | 128.5000 | 72.2500 |
| 0.0008 | – | -7.4167 | 30.8333 | 7.7500 | -42.6667 | 17.0000 | -27.0000 | -16.8333 | -28.6667 | -72.0000 | -62.0000 | -77.5833 | 27.1667 | -29.0833 |
| 0.0018 | *0.8053* | – | 38.2500 | 15.1667 | -35.2500 | 24.4167 | -19.5833 | -9.4167 | -21.2500 | -64.5833 | -54.5833 | -70.1667 | 34.5833 | -21.6667 |
| < 0.0001 | *0.3054* | *0.2036* | – | -23.0833 | -73.5000 | -13.8333 | -57.8333 | -47.6667 | -59.5000 | -102.8333 | -92.8333 | -108.4167 | -3.6667 | -59.9167 |
| 0.0003 | *0.7967* | *0.6141* | *0.4429* | – | -50.4167 | 9.2500 | -34.7500 | -24.5833 | -36.4167 | -79.7500 | -69.7500 | -85.3333 | 19.4167 | -36.8333 |
| 0.0512 | *0.1561* | *0.2413* | *0.0146* | *0.0938* | – | 59.6667 | 15.6667 | 25.8333 | 14.0000 | -29.3333 | -19.3333 | -34.9167 | 69.8333 | 13.5833 |
| < 0.0001 | *0.5720* | *0.4170* | *0.6456* | *0.7585* | *0.0473* | – | -44.0000 | -33.8333 | -45.6667 | -89.0000 | -79.0000 | -94.5833 | 10.1667 | -46.0833 |
| 0.0135 | *0.3694* | *0.5151* | *0.0545* | *0.2480* | *0.6025* | *0.1436* | – | 10.1667 | -1.6667 | -45.0000 | -35.0000 | -50.5833 | 54.1667 | -2.0833 |
| 0.0050 | *0.5758* | *0.7543* | *0.1131* | *0.4138* | *0.3905* | *0.2607* | *0.7354* | – | -11.8333 | -55.1667 | -45.1667 | -60.7500 | 44.0000 | -12.2500 |
| 0.0157 | *0.3406* | *0.4800* | *0.0479* | *0.2261* | *0.6417* | *0.1290* | *0.9558* | *0.6941* | – | -43.3333 | -33.3333 | -48.9167 | 55.8333 | -0.4167 |
| 0.3295 | *0.0167* | *0.0318* | *0.0006* | *0.0080* | *0.3295* | *0.0031* | *0.1347* | *0.0667* | *0.1497* | – | 10.0000 | -5.5833 | 99.1667 | 42.9167 |
| 0.1910 | *0.0393* | *0.0696* | *0.0020* | *0.0204* | *0.5204* | *0.0086* | *0.2446* | *0.1333* | *0.2678* | *0.7396* | – | -15.5833 | 89.1667 | 32.9167 |
| 0.4298 | *0.0099* | *0.0197* | *0.0003* | *0.0046* | *0.2458* | *0.0017* | *0.0927* | *0.0434* | *0.1039* | *0.8528* | *0.6045* | – | 104.7500 | 48.5000 |
| < 0.0001 | *0.3665* | *0.2503* | *0.9030* | *0.5186* | *0.0203* | *0.7354* | *0.0718* | *0.1436* | *0.0635* | *0.0010* | *0.0030* | *0.0005* | – | -56.2500 |
| 0.0163 | *0.3337* | *0.4714* | *0.0464* | *0.2208* | *0.6516* | *0.1256* | *0.9448* | *0.6839* | *0.9889* | *0.1537* | *0.2739* | *0.1069* | *0.0615* | – |
